# Dasatinib and quercetin prevent alveolar bone loss in aged mice

**DOI:** 10.1002/jper.70115

**Published:** 2026-03-26

**Authors:** Luisa S. Battistelli, Renata M. Moraes, Yuri C. Santos, Jaqueline L. Ribeiro, Maíra T. Garcia, Renata F. Prado, Florent Elefteriou, Ana Lia Anbinder

**Affiliations:** ^1^ Department of Biosciences and Oral Diagnosis, Institute of Science and Technology, São José dos Campos São Paulo State University (UNESP) São José dos Campos SP Brazil; ^2^ Center for Pediatric Neurological Disease Research St. Jude Children's Research Hospital Memphis Tennessee USA; ^3^ Department of Orthopedic Surgery Baylor College of Medicine Houston Texas USA

**Keywords:** aging, alveolar bone loss, cellular senescence, periodontitis

## Abstract

**Background:**

Recent findings have associated alveolar bone loss (ABL) with the presence of senescent cells. Aging and the accumulation of senescent cells are associated with a pro‐inflammatory environment which may exacerbate tissue damage. Senolytics, such as dasatinib and quercetin (D&Q), eliminate senescent cells, offering potential therapeutic benefits. We evaluated the effects of D&Q on ABL during physiological aging and experimental periodontitis.

**Methods:**

This study evaluated the effects of D&Q in two in vivo models: aging and ligature‐induced periodontitis. In the aging model, 24 male C57BL/6 mice were allocated to CTRL (control‐30 weeks of age), AGED (vehicle‐treated, euthanized after 10 months), or D&Q (monthly gavage of D&Q [5 mg/kg and 50 mg/kg] for 10 months). In the periodontitis model, 24 Wistar rats (12 weeks old) were divided into CTRL, EP (experimental periodontitis, ligature + *P. gingivalis*), or D&Q (EP + weekly D&Q at 5 mg/kg and 50 mg/kg). The ABL and bone microarchitecture were assessed by microcomputed tomography and histomorphometry, and gene expression was also analyzed. The microarchitecture of the femurs was evaluated for systemic effects.

**Results:**

In aged mice, D&Q reduced ABL, preserved the femoral cortical microarchitecture, and downregulated *Il6*, *Mmp13* and *Lamb1* expression. In contrast, D&Q failed to prevent ABL in the periodontitis model.

**Conclusion:**

D&Q treatment mitigated age‐related bone loss; but did not prevent EP associated bone loss under the therapeutic regimen used. These results suggest that the effects of senolytic therapy depend on disease context and treatment conditions.

**Plain language summary:**

Aging increases the risk of periodontitis due to immune decline, chronic inflammation, and the accumulation of senescent cells, which release inflammatory factors that damage tissues. Senolytic drugs, such as dasatinib and quercetin (D&Q), selectively eliminate senescent cells and have shown promise in age‐related diseases. In this study, we tested whether D&Q could reduce alveolar bone loss associated with aging or experimental periodontitis. The D&Q treatment protected aged mice by reducing bone loss, but it failed to prevent bone loss in the periodontitis model. These results suggest that the benefits of senolytic therapy may depend on disease context and treatment conditions.

## 1 INTRODUCTION

Several factors have been investigated to explain the link between aging and susceptibility to periodontal disease, based on epidemiological data indicating a significant increase in periodontitis prevalence and severity with aging.^1^, ^2^ Immunosenescence and inflammation may play a key role in the higher prevalence of inflammatory diseases in the elderly. The age‐related decline in immune cell function impairs tissue response and defense against pathogens, resulting in reduced resistance to infections.^3^, ^4^, ^5^

Cellular senescence is an integral part of the aging process and contributes to age‐related dysfunctions.^6^ It is triggered by various external and internal stressors and is characterized by an irreversible cell cycle arrest, cell enlargement, DNA damage, metabolic changes, and the release of pro‐inflammatory signals and proteases known as the senescence‐associated secretory phenotype (SASP), including interleukin (IL)‐1α, IL‐1β, IL‐6, and IL‐8.^6^, ^7^ These molecules primarily act as signals to neighboring immune cells, eventually facilitating the clearance of senescent cells. However, with aging, this balance is disrupted, leading to the accumulation of senescent cells in tissues and a state of chronic inflammatory stress due to persistent SASP secretion.^6^, ^7^

Alveolar bone loss (ABL) occurs with aging, associated with periodontal disease or not; however, the underlying biological mechanisms driving this age‐related susceptibility remain unclear. Osteocyte senescence in alveolar bone has been linked to age‐related ABL, and its early onset has been suggested as a potential risk factor for periodontitis.^8^, ^9^, ^10^ Aquino‐Martinez et al.^10^ reported an age‐related accumulation of senescent osteocytes in mouse alveolar bone, identifying them as a sustained source of inflammatory cytokines in vitro, which may contribute to the greater ABL observed in older mice. Additionally, senescence in alveolar bone osteocytes has been shown to occur earlier than in osteocytes from other mandibular regions, such as cortical and ramus bone.^9^ This site‐specific difference is believed to be associated with the unique characteristics of the alveolar bone and the periodontal environment, which lie in close proximity to the periodontal microbial biofilm and its toxin production. This suggests that exposure to bacterial genotoxins may drive premature senescence.^9^

SASP factors may contribute to the shift from symbiotic to pathogenic bacteria by exacerbating inflammation, altering tissue remodeling, and generating tissue breakdown products that promote pathogenic bacterial overgrowth.^8^, ^9^ Thus, there is good suggestive evidence in mice that senescence in alveolar bone contributes to the mechanisms driving inflammatory periodontal tissue destruction during aging and periodontal disease.

Selective elimination of senescent cells is a promising strategy to mitigate age‐related dysfunctions and chronic diseases. Several compounds have been identified that selectively induce senescent cell apoptosis both in vitro and in vivo, forming the basis of senolytic therapies.^11^ Senolytic agents have shown promising results in preclinical studies for treating age‐related conditions, including cardiac and metabolic dysfunction, and osteoporosis.^12^ Various classes of senolytic agents are under investigation, including dasatinib, quercetin and other Bcl‐2 family protein inhibitors, fisetin and piperlongumine.^12^, ^13^ The D&Q senolytic combination has been tested successfully in various animal models, demonstrating efficacy in reducing senescent cells associated with multiple diseases.^13^ In bone tissue, the combination D&Q has shown promising results in reducing age‐related systemic bone loss in mice.^14^ Together, this body of evidence led us to hypothesize that senolytic therapy (D&Q) could reduce both age‐related and periodontitis‐induced ABL. To test this hypothesis, we employed an aging mouse model and the ligature‐induced periodontitis rat model to test the putative beneficial effect of D&Q on ABL.

## 2 MATERIALS AND METHODS

This randomized and controlled experimental study was approved by the Institutional Animal Care and Use Committee of the Institute of Science and Technology, UNESP (IACUC approval numbers 07/2022 and 09/2023), and was conducted in accordance with ethical principles for animal experimentation. This study was designed following the ARRIVE guidelines.^15^ The experimental unit was a single animal. Simple randomization was performed using an online randomization tool.^16^ Animals were housed in groups of up to five per cage, with rodent laboratory diet* and water available ad libitum under a 12 h light/dark cycle. Blinding was applied only during outcome assessment and data analysis.

### 2.1 Aging mouse model

Twenty‐four male C57BL/6 mice (30 weeks old) were maintained under specific pathogen‐free (SPF) housing conditions and randomly assigned to three groups (*n* = 8): CTRL (control): euthanized at 30 weeks (age 7.5 months); AGED: received vehicle gavage monthly (200 µL) for 10 months (until 17.5 months of age); D&Q: received monthly gavage (200 µL) of dasatinib† (5 mg/kg) and quercetin‡ (50 mg/kg)^14^ and euthanized after 10 months (age 17.5 months). The monthly dosing regimen was selected based on the pharmacodynamics of senolytics, which do not require continuous drug exposure to exert their effects, and on prior protocols shown to be effective in chronic age‐related conditions characterized by slow senescent cell accumulation.^14^

Animals were weighed at the beginning of treatment and at the time of euthanasia. Power analysis indicated that an *n* = 8/group provided 80% power to detect a 0.03 mm (12%) difference in linear ABL.^17^, ^18^ The number of samples included in each analysis is indicated in the corresponding figure and table legends. Instances in which the final *n* was lower than 8 reflect technical issues rather than exclusion of animals, including sample loss or damage during processing, particularly during histological embedding and sectioning of small mouse specimens.

Drugs were diluted in 15% PEG400, 2% DMSO, 2.5% Tween 80, and 80.5% distilled water. The AGED group received vehicle. Euthanasia was performed by administering an intraperitoneal overdose of ketamine and xylazine, followed by decapitation. The right mandibles and femurs were fixed in 4% paraformaldehyde (PFA) and stored in 70% alcohol. The left mandibles, after alveolar bone dissection, were frozen immediately in liquid nitrogen for gene expression analysis.

#### 2.1.1 Microcomputed tomography (micro‐CT)

Scans were performed with a high‐resolution micro‐computed tomography system§ at 9.99 µm isotropic voxel size (9.9999 µm^3^), 70 kVp X‐ray tube potential, 142 µA source current, 1800 ms integration time, using an aluminum filter (0.5 mm), 0.5° rotation step, and frame averaging of 4. All images were reconstructed using dedicated reconstruction software∥ and reoriented using visualization software¶ for subsequent linear, area‐based, and volumetric analyses. Bone segmentation was performed using fixed global thresholds applied uniformly within each type of analysis, as follows: 70–115 for mandibular bone, 50–155 for cortical femoral bone, and 70–115 for medullary femoral bone analyses.

To measure the linear distance from the cementoenamel junction (CEJ) to the resorbed alveolar bone crest (ABC) at the lingual region and the area of the exposed lingual root surfaces of the lower molars, reoriented datasets were used to generate three‐dimensional (3D) models using 3D visualization software#, and the region of interest (ROI) corresponding to the entire fragment was selected. The CEJ‐ABC distance and the exposed lingual root surface area were measured using image analysis software**.

For volumetric and microarchitectural analyses, datasets of sagittal slices derived from the reconstructed images were analyzed using dedicated micro‐CT analysis software††. The mandible volume of interest (VOI) encompassed the alveolar bone surrounding the mandibular first molar and the interproximal alveolar bone between the first and second molars. The coronal limit was defined by the CEJ, and the apical limit by the coronal portion of the incisor periodontal ligament. The mesial boundary corresponded to the most mesial points of the alveolar bone adjacent to the first molar, whereas the distal boundary was defined by the periodontal ligament at the mesial aspect of the second molar.

Femurs were evaluated as an additional control for systemic effects. The analyzed VOI consisted of 100 CT scans of the trabecular/cortical bone at the distal metaphysis, starting 25 slices from the most proximal point of the growth plate.

The bone volume fraction (BV/TV), trabecular number (Tb.N), thickness (Tb.Th), separation (Tb.Sp), total porosity (Po.Tot), connectivity density (Conn.Dn), structural model index (SMI), as well as the cortical bone area (Ct.Ar), total cross‐sectional area (Tt.Ar), cortical area fraction (Ct.Ar/Tt.Ar), cortical bone volume (Ct.Bv), and thickness (Ct.Th) were calculated using standard methods.

#### 2.1.2 Histomorphometry

Mandibles were decalcified in 0.5 M ethylenediaminetetraacetic acid (EDTA) solution (pH 7.8), embedded in paraffin with the lingual surface facing downward, sectioned at 4 µm from the lingual to the buccal aspect in the sagittal plane, stained with hematoxylin and eosin (H&E), and photographed at 100×. The analysis included the linear distance and area between the interradicular septum and the cement surface in the first molar furcation region.

#### 2.1.3 Reverse transcription quantitative polymerase chain reaction (RT‐qPCR)

Tissues were processed using a phenol‐guanidine‐based reagent‡‡, RNA was extracted, treated with deoxyribonuclease§§, and 1 µg was reverse‐transcribed using a reverse transcription system∥∥. qPCR was performed using a DNA polymerase‐based detection system¶¶ on a real‐time PCR instrument##. The efficiency curves were generated using a cDNA pool containing samples from the AGED group, at the following concentrations: undiluted, 1:5, 1:25, 1:125, 1:625, and 1:3,125. Efficiency was calculated based on the slope of the standard curve, and all primers showed efficiencies within the acceptable range of 90%–110%. Reaction specificity was verified by melting curve analysis, confirming the presence of a single peak for each primer pair. Expression was calculated using the 2^−ΔΔCT^ method. After evaluating *Gapdh*, *Tbp*, and *Hprt* using a stability analysis algorithm***, *Hprt* was selected as the reference gene. Primer sequences are provided in Table S1 in the online *Journal of Periodontology*.

### 2.2 Ligature‐induced periodontitis model

Twenty‐four male adult Wistar rats weighing approximately 460 g (3 months old) were maintained under conventional housing conditions and randomly assigned to groups: CTRL (no induction), EP (experimental periodontitis, ligature + *Porphyromonas gingivalis*), D&Q (EP + weekly D&Q). Rats were chosen instead of mice for the periodontitis induction model because ligature placement is technically more feasible in rats. The weekly dosing regimen was selected based on the pharmacodynamics of senolytics, which do not require continuous drug exposure to exert their effects, and on a previously published protocol,^19^ while also accounting for the acute and rapidly evolving nature of ligature‐induced periodontitis, in which senescent cell accumulation may occur over a shorter timeframe than age‐related senescence.

Animals received sulfamethoxazole‐trimethoprim (53 mg/kg/day) in the drinking water for 10 days to reduce the resident microbiota, followed by 3 days of washout. Ligatures were placed around the lower first molars using a cotton thread††† (size 24) under intramuscular anesthesia with ketamine (100 mg/kg) and xylazine (5 mg/kg). Ligatures were checked after 15 days and repositioned into the gingival sulcus when necessary. *P. gingivalis* ATCC 33277 (10^9^ CFU in 100 µL of 2% carboxymethylcellulose) was administered orally eight times at 2‐day intervals (days 0 to 21).^20^ D&Q (5 mg/kg and 50 mg/kg) was administered by gavage on days 0, 7, 14, and 21.^19^ Animals were weighed before ligature placement and at the time of euthanasia. Euthanasia was performed on day 30. Tissues were fixed in 4% PFA and stored in 70% ethanol.

Power analysis indicated that *n* = 8/group was sufficient to detect 0.151 mm (13.72%) difference using linear ABL as the primary outcome.^18^, ^21^ The number of samples included in each analysis is indicated in the corresponding figure and table legends. Occasional discrepancies in group size (*n* < 8) resulted from technical losses during sample processing, including histological damage or ligature loss, rather than exclusion of animals.

#### 2.2.1 Microcomputed tomography

Mandibles were scanned using settings previously described. Bone segmentation was performed using fixed global thresholds (50–120). Bone loss was assessed via CEJ‐ABC distance and exposed root surface area. VOI was defined by lines touching the mesial, distal, buccal, and lingual roots of the first molar. The coronal limit was the furcation, and the apical limit was the last slice in which all four roots were visible.

#### 2.2.2 Histomorphometry

Left mandibles were decalcified, embedded, sectioned (5 µm), and stained with H&E, as described in Section 2.1.2. Three sections per animal were analyzed to assess the furcation ABL area.

### 2.3 Statistical analysis

Analysis of variance (ANOVA) or *t*‐tests were used for parametric data; Kruskal‐Wallis or Mann‐Whitney for non‐parametric. Significance was set at 5%. Analyses were performed using statistical analysis software‡‡‡.

## 3 RESULTS

### 3.1 Aging mouse model

Over the 10‐month observation period, several aging‐related changes were observed, including rectal prolapse and abnormal swelling near the genital region, consistent with seminal vesicle hyperplasia (Figure S1 in the online *Journal of Periodontology*). In addition, loss of fur shine in AGED mice compared with the CTRL and the treatment D&Q group was also noted (Figure S1).

There was no statistically significant difference in body weight between the CTRL group at the time of euthanasia and the AGED and D&Q groups at the beginning of treatment (7.5 months, ANOVA, *p* = 0.7375). Similarly, no significant difference was observed between the body weights of the AGED (17.5 months vehicle) and D&Q (17.5 months D&Q) groups at the time of euthanasia (Student's *t*‐test, *p* = 0.6427).

#### 3.1.1 D&Q prevents ABL and the deterioration of femoral cortical microarchitecture associated with aging

Through micro‐CT analyses and 3D reconstructions, dental misalignment was observed in aged mice (AGED and D&Q groups) compared with the CTRL group (Figure 1A–D), with the second and, predominantly, the third molars displaced lingually, slightly overlapping the first molar. Additionally, pronounced occlusal wear was noted in aged mice, characterized by flattened cusps, dentin exposure, and reactive dentin deposition.

**FIGURE 1:**
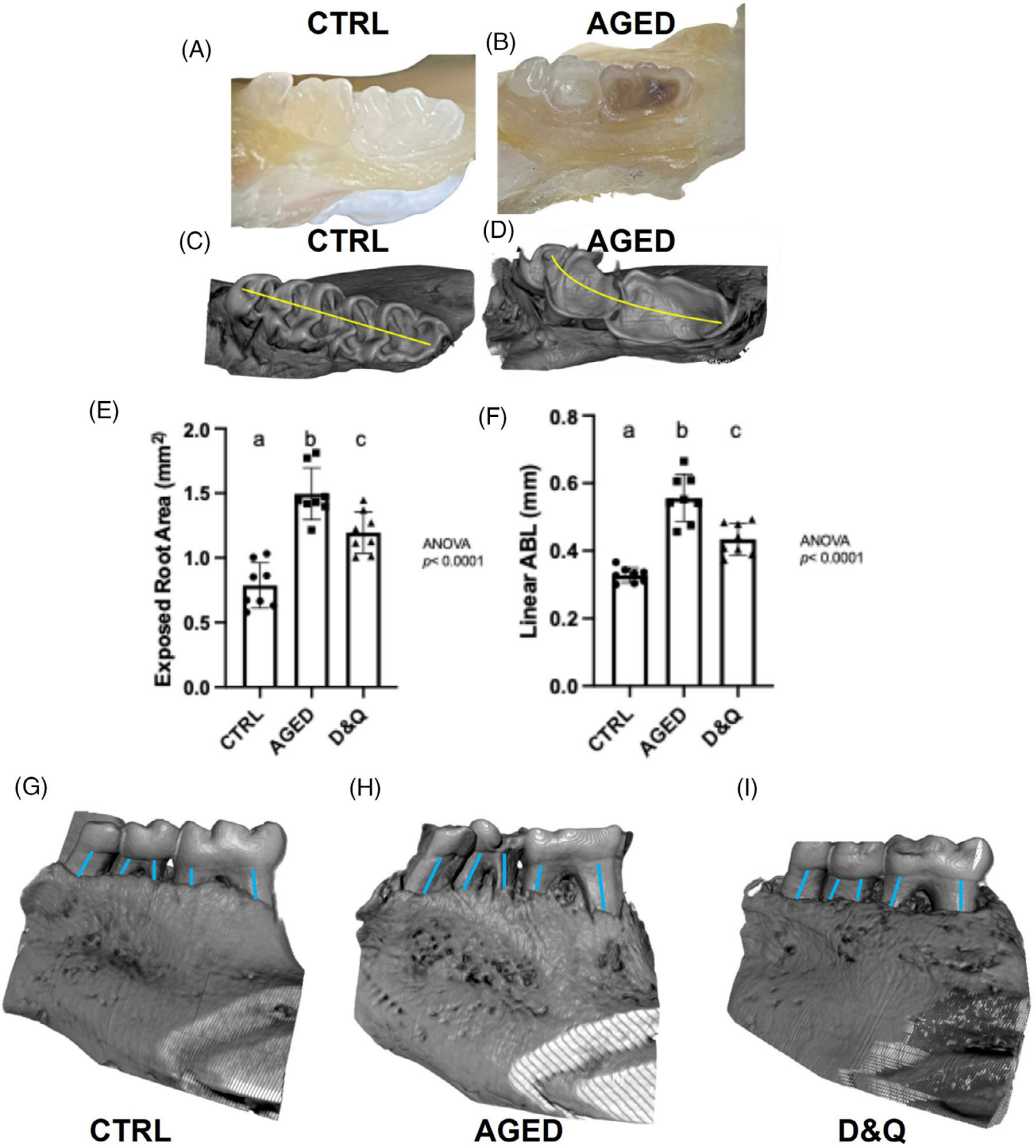
Dental alterations and alveolar bone loss. (A) Macroscopic view of the mandible from a CTRL mouse, showing aligned teeth with well‐preserved cusps. (B) Macroscopic view of the mandible from an AGED mouse, exhibiting dental misalignment with lingual curvature, worn cusps, and darkened occlusal surfaces (reparative dentin) on the first molar. (C) 3D model of a CTRL mouse showing aligned teeth with preserved cusps. (D) 3D model of an AGED mouse displaying dental misalignment due to lingual curvature and tooth movement, along with cusp wear. Bar graphs with individual data points (mean ± SD) showing the exposed lingual root surface area (E) and the linear distance from the CEJ to the ABC (F) of the lower molars. Different lowercase letters above the bars indicate statistically significant differences (*p* < 0.05). (G–I) 3D reconstructions of the mandible from representative mice in the CTRL group (G), AGED group (H), and D&Q group (I) (*n* = 8 mice/group). Blue lines indicate the linear distance from the cementoenamel junction (CEJ) to the resorbed alveolar bone crest (ABC).

Aging in the AGED group resulted in a significant increase in the CEJ‐ABC distance and the exposed lingual root surface area of the lower molars compared with the CTRL group. Treatment with D&Q significantly reduced ABL compared with the AGED group, though it did not fully restore values to those observed in the CTRL group (Figures 1E–I).

In the VOI delineated in the sagittal plane, the AGED group exhibited a significant decrease in BV/TV and Tb.N and an increase in Po(tot) compared with CTRL. Treatment was sufficient to restore Tb.N to the level of the CTRL group, although it also remained statistically similar to the AGED group (Table 1).

**TABLE 1:** Microarchitectural parameters in alveolar bone.

Aging mouse model					
Parameter	CTRL	AGED	D&Q	Statistical test	*p*‐value
BV/TV (%)	25.81 ± 2.16^a^	19.43 ± 4.77^b^	19.71 ± 4.48^b^	ANOVA	0.0061
Tb.Th (mm)	0.082 ± 0.004	0.076 ± 0.006	0.077 ± 0.008	ANOVA	0.1485
Tb.N (1/mm)	3.338 ± 0.374^a^	2.595 ± 0.703^b^	2.877 ± 0.489^ab^	ANOVA	0.0374
Tb.Sp (mm)	0.124 ± 0.008	0.139 ± 0.021	0.134 ± 0.017	ANOVA	0.1725
Conn.Dn (1/mm^3^)	230.6 ± 36.14	240.3 ± 73.96	215.6 ± 27.06	ANOVA	0.6159
Po.Tot (%)	74.19 ± 2.16^a^	80.57 ± 4.77^b^	80.29 ± 4.48^b^	ANOVA	0.0061

Ligature‐induced periodontitis rat model					
Parameter	CTRL	EP	D&Q	Statistical test	*p*‐value
BV/TV (%)	76.4 (62.65–78.05)^a^	56.92 (38.9–61.3)^b^	55.08 (38.55–63.84)^b^	Kruskal‐Wallis	0.0014
Tb.Th (mm)	0.207 ± 0.064^a^	0.151 ± 0.019^b^	0.177 ± 0.022^ab^	ANOVA	0.0398
Tb.N (1/mm)	3.757 ± 1.085	3.043 ± 0.667	3.062 ± 0.361	ANOVA	0.1392
Tb.Sp (mm)	0.095 (0.078–0.118)^a^	0.1615 (0.13–0.407)^b^	0.1925 (0.140–0.352)^b^	Kruskal‐Wallis	0.0008
Conn.Dn (1/mm^3^)	56.9 ± 17.21	60.91 ± 20.42	65.02 ± 39.16	ANOVA	0.8674
Po.Tot (%)	23.6 (21.95–37.35)^a^	43.08 (38.7–61.08)^b^	44.92 (36.16–61.45)^b^	Kruskal‐Wallis	0.0014

Different superscript letters within a row indicate statistical significance (*p* < 0.05). Parametric data are presented as mean ± SD, while non‐parametric data are presented as median (minimum‐maximum). *n* = 7–8 animals/group, except for Conn.Dn in the CTRL group of the periodontitis model, where *n* = 6 rats/group.

The AGED group exhibited a greater distance between the cementum and the top of the ABC in the furcation region (Figure 2). Treatment was sufficient to prevent the linear furcation ABL to levels comparable to the CTRL group, although no significant differences were observed between the groups for the area of bone loss/periodontal ligament space in the interradicular region (Figure 2).

**FIGURE 2:**
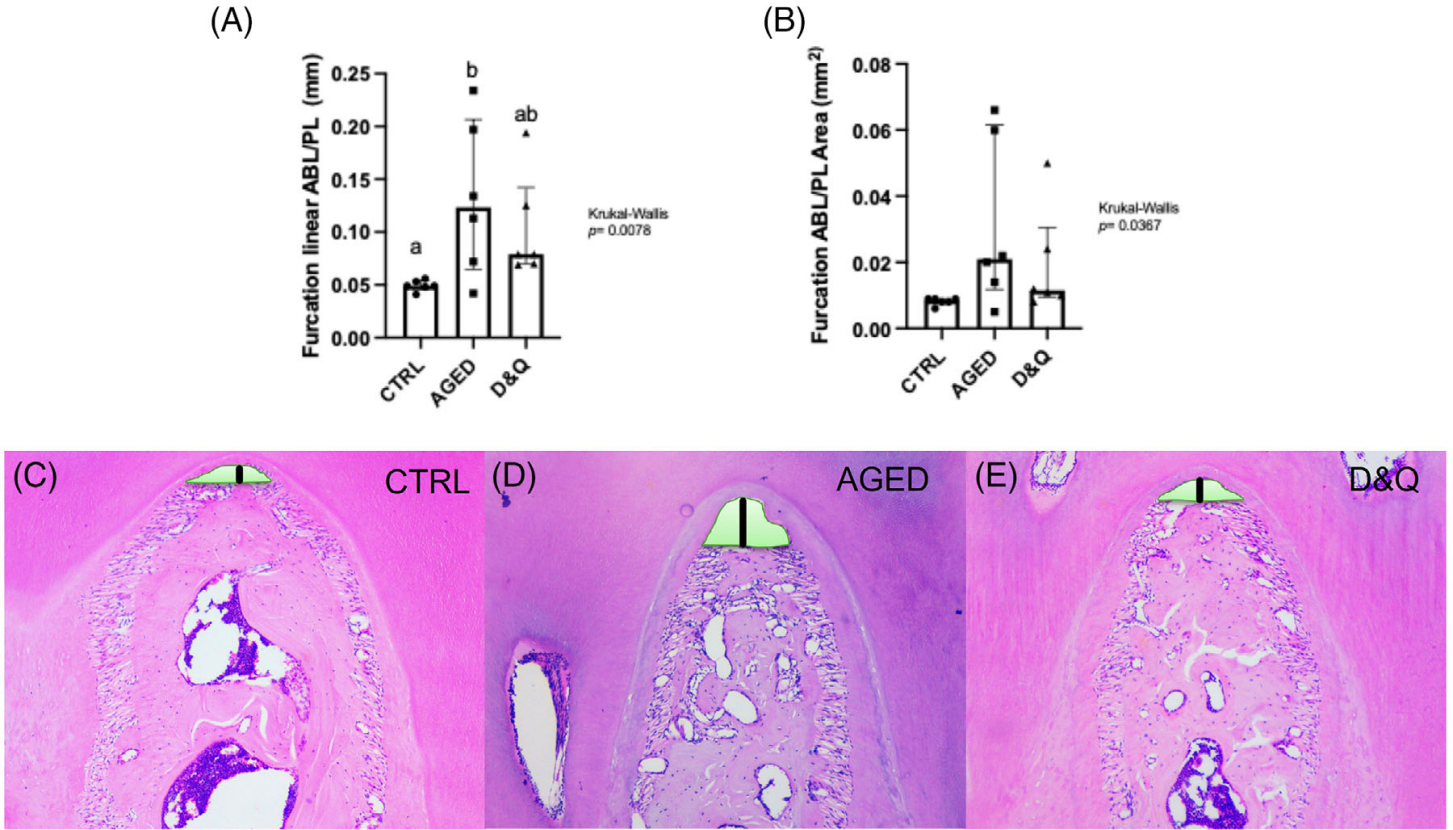
Median and interquartile range plots with individual data points of histomorphometric analyses: (A) linear bone loss/periodontal ligament space in the interradicular region; and (B) area of bone loss/periodontal ligament space in the interradicular region. Kruskal–Wallis test showed overall significance (*p* < 0.05), but post hoc multiple comparisons revealed no statistically significant differences between groups. *n* = 6 mice/group. Photomicrographs of the first molar furcation region from representative mice in the CTRL group (C), AGED group (D), and D&Q group (E). The black line indicates linear furcation ABL/periodontal ligament, and the green shaded area highlights the furcation ABL/periodontal ligament area. H&E staining. Original magnification ×100.

Aging adversely affected femoral trabecular microarchitectural parameters regardless of treatment, with the exception of Tb.Th and SMI, for which D&Q treatment exerted a positive effect (Table S2 in the online *Journal of Periodontology*). The treatment was also effective in preventing the age‐related reduction in Ct.Ar/Tt.Ar, Ct.BV, and Ct.Th (Figure 3). These results are consistent with previous reports,^14^ confirm drug activity in our aging model and impact on both alveolar and appendicular bones.

**FIGURE 3:**
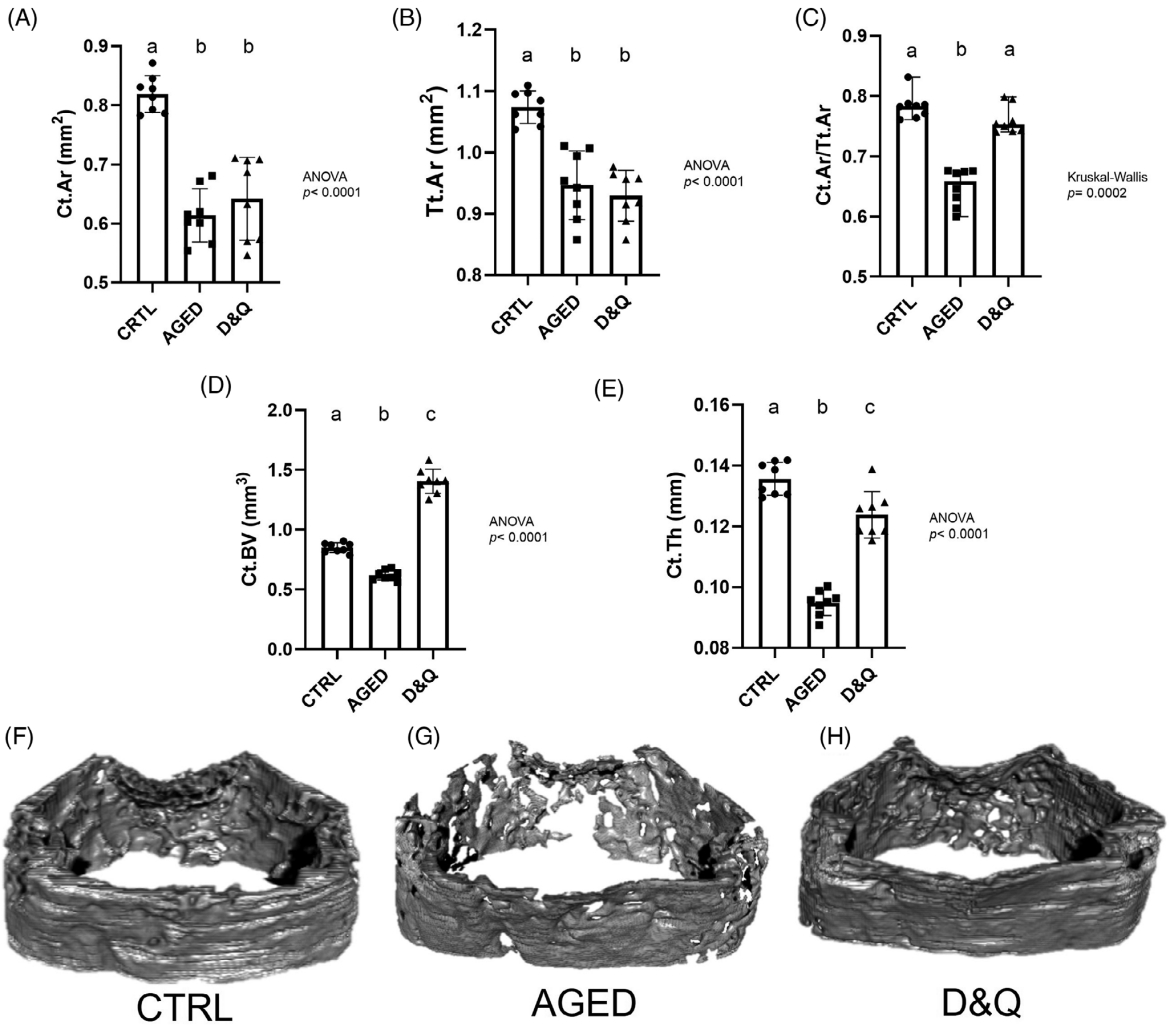
Mean and standard deviation (A, B, D, E) and median with interquartile range (C) plots with individual data points showing cortical femoral microarchitectural parameters: (A) Ct.Ar (cortical bone area), (B) Tt.Ar (total cross‐sectional area), (C) Ct.Ar/Tt.Ar (cortical area fraction), (D) Ct.BV (cortical bone volume), and (E) Ct.Th (cortical thickness). Different lowercase letters above the bars indicate statistically significant differences (*p* < 0.05). *n* = 8 mice per group. Representative 3D models of the femoral cortex from the CTRL (F), AGED (G), and D&Q (H) groups.

#### 3.1.2 D&Q reduces alveolar bone gene expression of *Il6, Mmp1*3, and *Lamb1* increased due to aging

Treatment with D&Q significantly reduced the age‐related increase in the alveolar bone expression of *Il6*, *Lamb1*, and *Mmp13* (Figure 4). The treatment showed a trend toward reducing *P21* expression; however, this difference did not reach statistical significance after multiple comparison testing.

**FIGURE 4:**
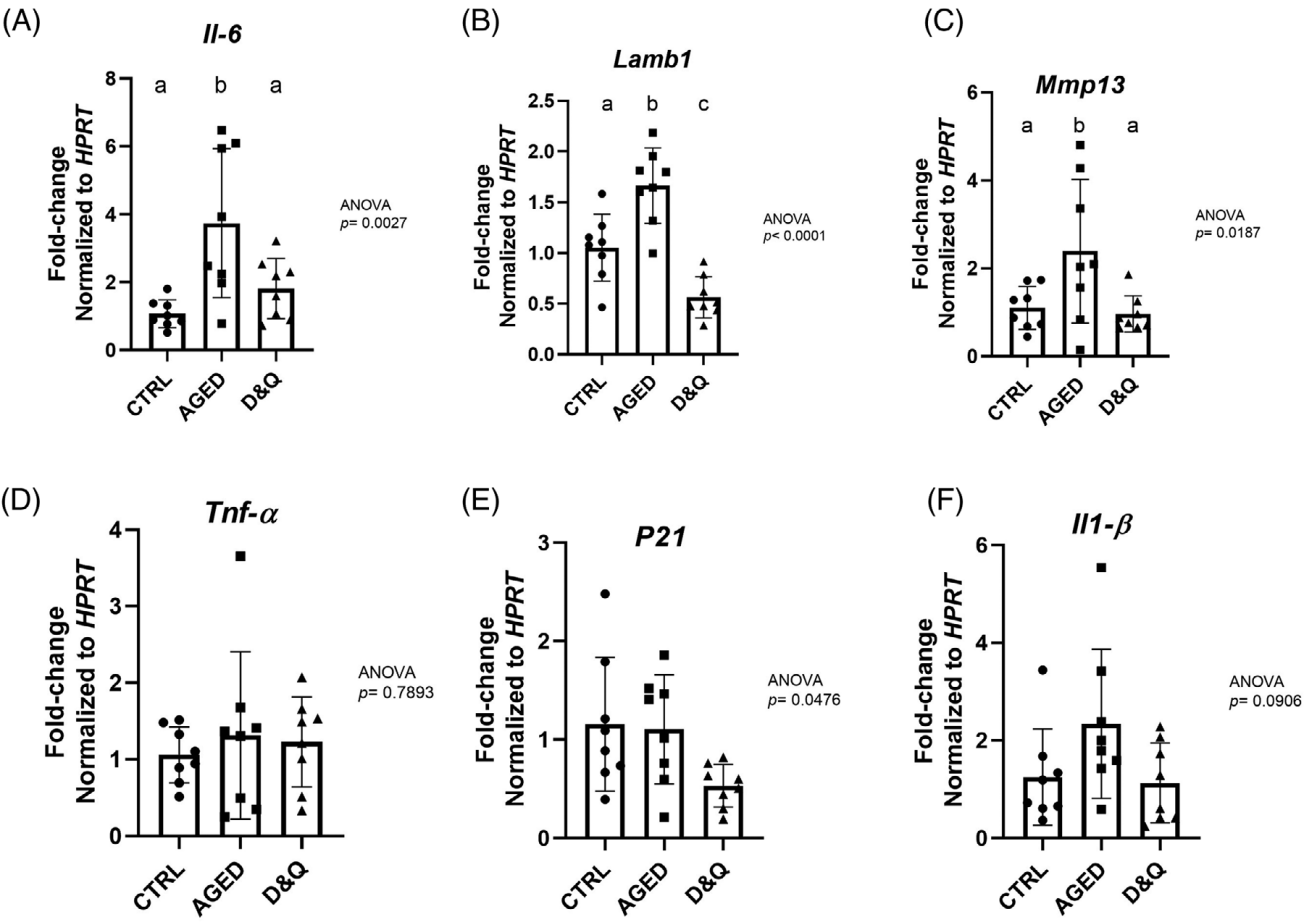
Mean and standard deviation graphs with individual data points of normalized expression of *Il‐6* (A), *Lamb1* (B), *Mmp13* (C), *Tnf‐α* (D), *P21* (E), *Il‐1β* (F). Different lowercase letters above the bars indicate statistical significance (*p* < 0.05). *n* = 8 mice/group.

### 3.2 Ligature‐induced periodontitis model

No significant differences in body weight were observed between groups at ligature placement (ANOVA, *p* = 0.3547) or at euthanasia (ANOVA, *p* = 0.9717).

#### 3.2.1 Weekly D&Q does not prevent ABL due to induced periodontitis

Both EP and D&Q groups showed increased CEJ‐ABC distances, greater exposed root areas in 3D reconstructions, and enhanced ABL in the furcation region on histology compared with CTRL, with no significant differences between EP and D&Q (Figure 5). These groups also exhibited reduced BV/TV, Po.Tot, and Tb.Sp compared with CTRL. Although the D&Q group showed a trend toward increased Tb.Th compared with vehicle, this was not statistically significant (Table 1). These results confirm the effectiveness of the experimental model in inducing bone loss, while the treatment was not effective in preventing it.

**FIGURE 5:**
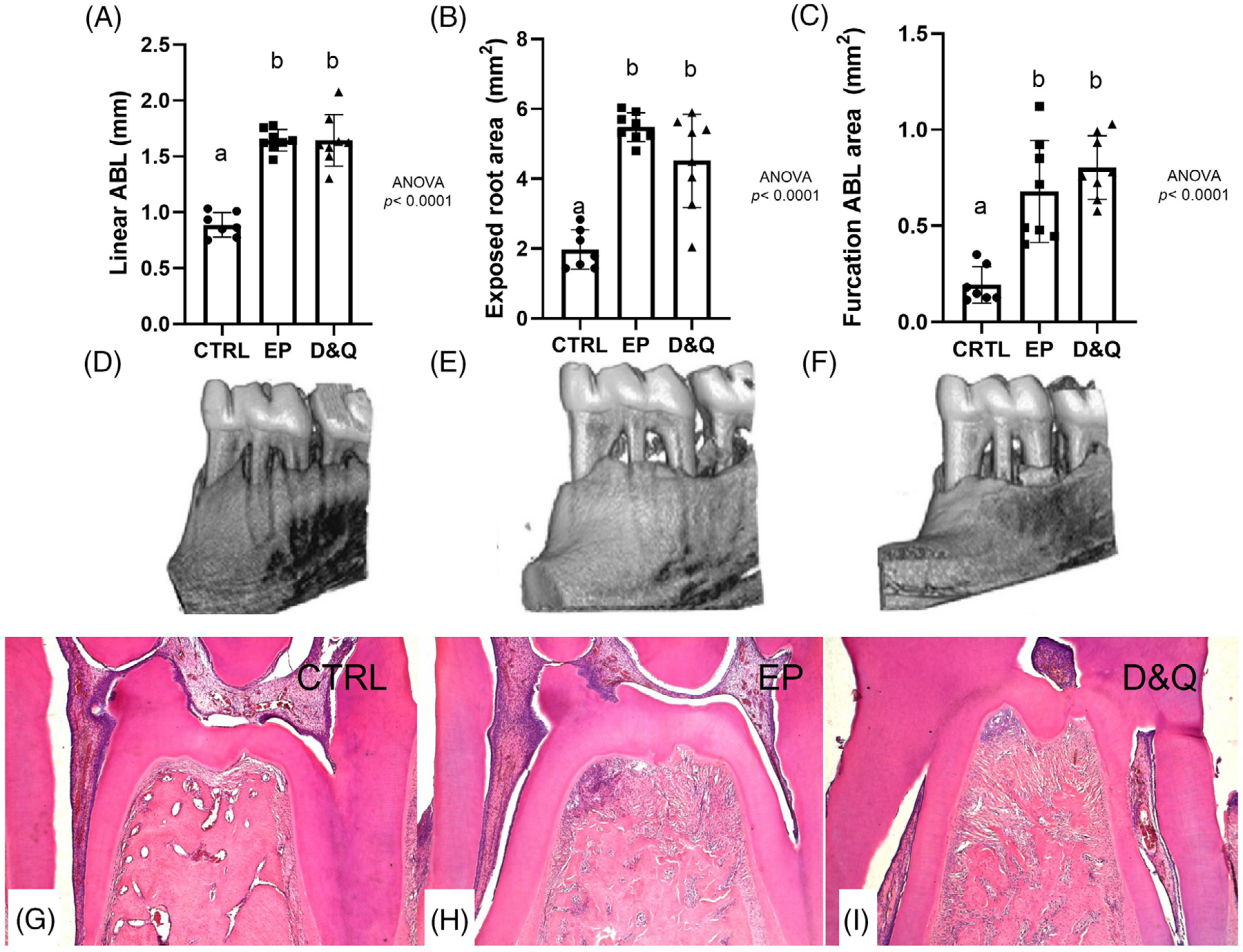
Mean and standard deviation graphs with individual data points of the linear distance from the CEJ to the ABC (Linear alveolar bone loss) (A), of the exposed lingual root surface area of first molar (B), and of furcation ABL area on histology (C) (*n* = 7–8 rats/group). Different letters above the bars indicate statistically significant differences (*p* < 0.05). 3D models of the first molar from representative rats in the CTRL (D), EP (E), and D&Q (F) groups. Photomicrographs of the first molar furcation region from representative rats in the CTRL (G), EP (H), and D&Q (I) groups. H&E staining. 50× Original magnification.

## 4 DISCUSSION

In this study, 30‐week‐old mice were treated with the combination D&Q or vehicle monthly for 10 months. During this long‐term treatment, aging‐related signs such as rectal prolapse, and seminal vesicle enlargement were observed. These findings are consistent with the literature^22^ and underscore the challenges of long‐term studies, including animal loss that may affect data integrity. In addition, occlusal wear was evident in aged mice. A 75% reduction in cusp height with preserved cusp bases in mice up to 100 weeks old has been reported.^17^ In contrast, the present study showed more pronounced wear reaching the cusp bases with dentin exposure, consistent with previous findings.^23^ These differences may be related to variations in mouse strain and diet.

Both root exposure area and the distance from the ABC to the CEJ were significantly higher in aged versus young mice, consistent with previous reports in mice.^10^, ^23^, ^24^, ^25^, ^26^ While age‐related bone loss in other bones is well documented,^27^ the existence of age‐associated ABL in humans remains debated. Still, several studies have reported increased ABL in elderly individuals without periodontitis,^26^ and a significant association between oral bone resorption and age has been observed, with minimal contribution from oral hygiene.^28^ Recent data also show greater bone loss and reduced bone volume in older individuals with healthy periodontium.^29^ Although the mechanisms underlying physiological ABL remain unclear, it is currently thought they involve disrupted host–microbiota balance,^30^ along with the accumulation of senescent osteocytes, reduced osteocyte numbers, increased cellular senescence and neutrophil infiltration.^8^, ^10^, ^31^

Following treatment with D&Q, a statistically significant reduction in the ABL and improvement of cortical femur microarchitecture were observed compared with untreated aged mice. Previous studies have demonstrated the effectiveness of D&Q in reducing senescent cell burden and improving bone structure in aged mice. Monthly D&Q treatment from 20 to 24 months of age significantly decreased p16^Ink4a^ expression and senescent osteocyte accumulation, resulting in improved trabecular bone microarchitecture in the spine and femur.^14^ Notably, this therapeutic effect was not observed in younger adult mice (12 months old), indicating that senolytics acted specifically in the context of age‐related bone deterioration. Similarly, recent studies reported that short‐term D&Q treatment (6 to 12 weeks) in 15‐ to 18‐month‐old mice reduced bone and gingival SASP expression, and attenuated naturally progressing periodontal bone loss.^31^, ^32^ These findings supported the potential of targeting senescence in aged individuals to promote alveolar bone health.

In contrast to these studies, we adopted a preventive strategy by initiating D&Q treatment earlier, at 7 months of age, and continuing it monthly for 10 months. This extended regimen led to significant reductions in ABL and improved femoral cortical microarchitecture, indicating that long‐term senolytic intervention starting in early adulthood can confer protective, whole body skeletal effects.

The discrepancy between mandibular lingual linear ABL in 3D reconstruction and microarchitectural outcomes likely reflects differences in measurement sensitivity. While senolytic treatment resulted in significant improvements in linear and area‐based parameters, which capture changes at the highly dynamic alveolar crest, no corresponding effects were detected in microarchitectural parameters, which represent an average across the entire VOI and may therefore dilute localized treatment effects.

An unanticipated, qualitative finding was the maintenance of fur luster in aged animals treated with D&Q, which was visibly improved compared with the AGED group. Although not quantitatively assessed in this study, this observation parallels findings reported by other authors^31^ and further supports the notion that senolytic therapy may exert systemic benefits beyond skeletal outcomes.

Chu et al.^33^ demonstrated that clearance of p16^INK4a^‐positive senescent cells using the P16‐3MR transgenic mouse model and daily ganciclovir treatment significantly reduced inflammation and bone loss during experimental periodontitis, suggesting a direct causal link between senescence and disease pathology. Similarly, Tang et al.^34^ showed that continuous dietary supplementation with the antioxidant pyrroloquinoline quinone, for 2 weeks during periodontitis induction, mitigated oxidative stress and reduced expression of senescence‐related markers such as p16 and p21 in periodontal mouse tissues, highlighting the benefit of sustained modulation of redox and senescence pathways. In our study, weekly administration of D&Q was not sufficient to prevent rat ABL in the ligature‐induced periodontitis model, in contrast with these previous studies in which continuous or more frequent senolytic regimens yielded favorable outcomes. More recently, Wang et al.^35^ reported that administration of D&Q on days 0 and 3 after ligature placement attenuated ABL at 7 days, indicating that shortening the initial treatment interval may enhance therapeutic efficacy. Considering that the greatest ABL in ligature‐induced periodontitis occurs within the first 2 weeks,^36^ early and repeated dosing during this critical window appears to be essential. Thus, the lack of additional doses in the initial phase may explain why our regimen of weekly administration was not sufficient to prevent ABL after 30 days, underscoring that the timing of senolytic delivery may be as decisive as the overall duration of treatment.

The optimal dosing strategy for senolytic therapy remains an open question. Khosla et al.^37^ emphasized that intermittent rather than continuous administration of senolytics may better preserve tissue healing capacity, particularly in skin and lung injury models. Continuous dosing has the potential to impair reparative responses, while intermittent dosing may strike a “therapeutic sweet spot”. Therefore, although our weekly administration may have been insufficient in the acute periodontitis context, these findings reinforce the need for model‐specific, optimized senolytic regimens that balance efficacy with preservation of physiological senescence‐driven repair mechanisms.

Aged mice exhibited elevated *Il‐6* expression in alveolar bone, in agreement with previous findings.^38^ *P21*, a cyclin‐dependent kinase inhibitor involved in cell cycle arrest, is commonly upregulated during cellular senescence.^39^ However, in line with our results, no significant differences in *P21* gene expression were observed between age groups in a prior study evaluating periodontal ligament tissues.^38^ Matrix metalloproteinases, particularly *Mmp13*, are also SASP components and are typically secreted at higher levels during senescence.^39^ An increase in *Mmp13* expression in osteocytes from the alveolar bone of aged mice has been reported previously,^10^ aligning with our findings. Notably, senolytic treatment significantly reduced *Mmp13* expression to levels comparable to young controls, suggesting its potential to suppress SASP‐related tissue degradation.

Laminins are a family of extracellular glycoproteins essential to the structural organization of basement membranes in nearly all tissues. A previous study reported elevated laminin levels in gingival crevicular fluid of untreated periodontitis patients, which may reflect increased neutrophil activity during transmigration.^40^ Similarly, aged animals with greater ABL in our study showed increased laminin expression, which was reduced following D&Q treatment, further supporting its modulatory effect on inflammation‐associated matrix remodeling.

Although our findings indicate that D&Q modulates inflammatory and senescence‐associated gene expression, our study has some limitations. We focused on a selected panel of SASP‐related pro‐inflammatory markers (*Il‐6*, *Il‐1β*, *Mmp13*, and *Tnf‐α*), which provide insight into the local inflammatory environment. However, we did not evaluate other key features of cellular senescence beyond *P21*. A more comprehensive analysis of these parameters would strengthen the interpretation of senescence dynamics in the context of alveolar bone. Integrating broader senescence profiling and microbial community analyses in future studies will be essential to fully understand the interplay between cellular aging, inflammation, and tissue breakdown in periodontal disease.

## 5 CONCLUSION

In conclusion, our findings demonstrate that long‐term monthly administration of D&Q, initiated in early adulthood, can attenuate age‐related ABL in mice. While the senolytic treatment also modulated the expression of key pro‐inflammatory genes in aged alveolar bone, the weekly regimen was not effective in preventing bone loss in the ligature‐induced periodontitis rat model, indicating that the therapeutic efficacy of D&Q in the context of ABL may depend on disease context, timing, treatment frequency and duration. These results support the potential of senescence‐targeted strategies as preventive interventions for age‐associated ABL.

## AUTHOR CONTRIBUTIONS

**Luisa S. Battistelli**: Methodology; formal analysis; data curation; investigation; writing—original draft; writing—review and editing; visualization. **Renata M. Moraes**: Conceptualization; validation; writing—original draft; writing—review and editing. **Yuri C. Santos**: Methodology; data curation; investigation; writing—review and editing. **Jaqueline L. Ribeiro**: Investigation; methodology; data curation; writing—review and editing. **Maíra T. Garcia**: Investigation; writing—review and editing. **Renata F. Prado**: Investigation; formal analysis; data curation; writing—review and editing. **Florent Elefteriou**: Conceptualization; validation; visualization; writing—original draft; writing—review and editing. **Ana Lia Anbinder**: Conceptualization; funding acquisition; methodology; investigation; formal analysis; visualization; writing—original draft; writing—review and editing; validation; project administration; supervision. All authors reviewed and approved the final manuscript and agreed to be accountable for all aspects of the work.

## CONFLICT OF INTEREST STATEMENT

The authors declare that they have no conflicts of interest.

## ETHICS STATEMENT

This experimental study was approved by the Institutional Animal Care and Use Committee of the Institute of Science and Technology, UNESP (IACUC approval numbers 07/2022 and 09/2023).

## Supporting information



Supporting Information

Supporting Information

## Data Availability

The data that support the findings of this study are available from the corresponding author upon reasonable request.

## Footnotes

* Autoclavable pelleted rodent laboratory diet (Ratos e Camundongos Peletizado Autoclavável), Neovia Nutrição e Saúde Animal Ltda, Descalvado, Brazil

† Merck Sigma‐Aldrich, St. Louis, MO, USA

‡ Merck Sigma‐Aldrich, St. Louis, MO, USA

§ SkyScan 1272, Bruker, Kontich, Belgium

^∥^
Nrecon Version 1.7.4.6, Bruker MicroCT, Kontich, Belgium

^¶^
DataViewer Version 1.5.1.2, Bruker MicroCT, Kontich, Belgium

# CTVox Version 3.0.0 r1114, Bruker MicroCT, Kontich, Belgium

** ImageJ, National Institutes of Health, Bethesda, MD, USA

†† CT Analyzer (CTAn), Version 1.15.4.0, Bruker MicroCT, Kontich, Belgium

‡‡ TRIzol, Invitrogen, Carlsbad, CA, USA

§§ DNase I, Promega, Madison, WI, USA

^∥∥^
GoScript Reverse Transcription Mix, Random Primers, Promega, Madison, WI, USA

^¶¶^
GoTaq RT‐qPCR Systems, Promega, Madison, WI, USA

## StepOnePlus, Applied Biosystems, Foster City, CA, USA

*** NormFinder, MOMA – Department of Molecular Medicine, Aarhus University Hospital, Aarhus, Denmark

††† Coats Corrente, São Paulo, Brazil

‡‡‡ GraphPad Prism, version 10.2, GraphPad Software, San Diego, CA, USA
