# Supplementary material for: Dasatinib and quercetin prevent alveolar bone loss in aged mice
Source: J Periodontol. 2026 Mar 26;97(10):2160–72. doi: 10.1002/jper.70115 (PMC13743505; doi:10.1002/jper.70115)
Supplement: Supplementary file 1 [file JPER-97-2160-s001.docx]

**SUPPLEMENTARY MATERIAL**

**Table S1**. Primer sequences for RT-qPCR reaction.

| Gene |  | Sequence |
| --- | --- | --- |
| *Hprt* | Forward  Reverse | *TCAGTCAACGGGGGACATAAA*  *GGGGCTGTACTGCTTAACCAG* |
| G*apdh* | Forward  Reverse | *AGGTCGGTGTGAACGGATTTG*  *TGTAGACCATGTAGTTGAGGTCA* |
| *Tbp* | Forward  Reverse | *CCTTGTACCCTTCACCAATGAC*  *ACAGCCAAGATTCACGGTAGA* |
| *Il-6* | Forward  Reverse | *TAGTCCTTCCTACCCCAATTTCC*  *TTGGTCCTTAGCCACTCCTTC* |
| *Tnfα* | Forward  Reverse | *CCCTCACACTCAGATCATCTTCT*  *GCTACGACGTGGGCTACAG* |
| *Il1-β* | Forward  Reverse | *GGAGAACCAAGCAACGACAAAATA*  *TGGGGAACTCTGCAGACTCAAAC* |
| *P21* | Forward  Reverse | *CCTGGTGATGTCCGACCTG*  *CCATGAGCGCATCGCAATC* |
| *Mmp13* | Forward  Reverse | *CAGTTGACAGGCTCCGAGAA*  *TTCACCCACATCAGGCACTC* |
| *Lamb1* | Forward  Reverse | *ATGGGTGTCGACCTTGTGAC*  *TTACACTGCCGCCCAATCAT* |

# **Table S2**. Femur trabecular microarchitectural parameters

| Parameter | CTRL (mean ±SD) | AGED (mean ±SD) | D&Q (mean ±SD) |
| --- | --- | --- | --- |
| BV/TV (%) | 13.170 ± 1.340^a^ | 3.045 ± 1.485^b^ | 3.554 ± 0.677^b^ |
| Tb.Th (mm) | 0.036 ± 0.002^a^ | 0.037 ± 0.001^a^ | 0.043 ± 0.002^b^ |
| Tb.N (1/mm) | 3.620 ± 0.454^a^ | 0.818 ± 0.358^b^ | 0.869 ± 0.182^b^ |
| Tb.Sp (mm) | 0.261 ± 0.015^a^ | 0.413 ± 0.053^b^ | 0.423 ± 0.026^b^ |
| Conn.Dn (1/mm^3) | 241.900 ± 34.630^a^ | 116.100 ± 27.610^b^ | 107.300 ± 14.450^b^ |
| SMI | 0.806 ± 0.277^a^ | 2.423 ± 0.239^b^ | 2.074 ± 0.173^c^ |
| Po.Tot (%) | 86.830 ± 1.340^a^ | 96.950 ± 1.485^b^ | 96.450 ± 0.677^b^ |

Different superscript letters within a row indicate statistical significance based on ANOVA followed by Tukey’s multiple comparison test (p < 0.0001).

**Figure S1**. Aging related changes. A) Abnormal swelling near the genital region; B) Seminal vesicle hyperplasia; C) Rectal prolapse; loss of fur shine in AGED animals (E) compared to the CTRL (D) and D&Q group (F).
